# In-House Immunoglobulin Y-Based Immunoassay for Detecting Benzo[a]pyrene in Grilled Pork Samples

**DOI:** 10.3390/bios14120588

**Published:** 2024-12-02

**Authors:** Peerapong Jeeno, Sumed Yadoung, Marninphan Thongkham, Pichamon Yana, Udomsap Jaitham, Sakaewan Ounjaijean, Zhen-Lin Xu, Korawan Sringarm, Surat Hongsibsong

**Affiliations:** 1 School of Health Sciences Research, Research Institute for Health Sciences, Chiang Mai University, Chiang Mai 50200, Thailand; peerapong_jeen@cmu.ac.th (P.J.); pichamon.y@cmu.ac.th (P.Y.); udomsap_j@cmu.ac.th (U.J.); sakaewan.o@cmu.ac.th (S.O.); 2 Environmental, Occupational Health Sciences and NCD Center of Excellence, Research Institute for Health Sciences, Chiang Mai University, Chiang Mai 50200, Thailand; sumed_y@cmu.ac.th; 3 Environmental Science Program, Faculty of Sciences, Chiang Mai University, Chiang Mai 50200, Thailand; 4 Department of Animal and Aquatic Sciences, Faculty of Agriculture, Chiang Mai University, Chiang Mai 50200, Thailand; marninphan_t@cmu.ac.th (M.T.); korawan.s@cmu.ac.th (K.S.); 5 Guangdong Provincial Key Laboratory of Food Quality and Safety, College of Food Science, South China Agricultural University, Guangzhou 510642, China; jallent@163.com

**Keywords:** Benzo[a]pyrene, immunoglobulin Y, immunoassay, ELISA, PAHs

## Abstract

Benzo[a]pyrene (B[a]P) is a hazardous polycyclic aromatic hydrocarbon that accumulates in several environmental matrices as a result of incomplete combustion. Its presence, carcinogenic properties, and tendency for bioaccumulation provide significant risks to human health and the environment. The objective of this study is to create an immunoassay for the detection of benzo[a]pyrene utilizing immunoglobulin Y antibodies. An indirect competitive enzyme-linked immunosorbent assay (ic-ELISA) was utilized to develop a speedy, straightforward, sensitive, and economical approach for detecting B[a]P residues. Following the immunization of hens with the hapten pyrenebutyric acid-bovine serum albumin (PyBA-BSA), the IgY antibody extracted from egg yolk was utilized to identify B[a]P residues. To evaluate antibody specificity, six PAH derivatives—PyBA, B[a]P, Chrysene, Benzo[b]fluoranthene, Benzo[a]anthracene, and Benzo[k]fluoranthene—were examined in the experiment to compete for binding with PyBA. The findings indicate that the antibody had considerable affinity for Chrysene (1.15%), Benzo[b]fluoranthene (311.32%), Benzo[k]fluoranthene (10.62%), Benzo[a]anthracene (22.82%), and PyBA (9.55%). Nonetheless, its affinity for B[a]P remained at 100%. The recovery range for grilled pork samples spiked with B[a]P doses of 10.00–0.1 μg/mL was 74.99% to 143.11%. This study utilized a polyclonal antibody, employing the IgY antibody for the inaugural development of an immunoassay to detect benzo[a]pyrene. The ELISA had a higher IC_50_ value compared to the other immunoassays; however, it yielded good results. This immunoassay signifies a substantial progression in environmental analytical chemistry, offering a cost-effective and accessible technique for the detection of B[a]P to protect human health and the environment.

## 1. Introduction

Benzo[a]pyrene (B[a]P) is a polycyclic aromatic hydrocarbon (PAH) produced by the incomplete combustion of organic materials at temperatures ranging from 300 to 600 °C. It is benzopyrene, formed by the fusion of a benzene ring with pyrene. B[a]P is present in various sources, such as coal tar, tobacco smoke, and numerous foods, especially grilled meats [1]. This chemical is well-known for its carcinogenic properties, as its metabolites are mutagenic and exceedingly carcinogenic, and it has been classified as a Group 1 carcinogen by the International Agency for Research on Cancer. The compound is chemically characterized by a cyclopentapyrene ring fused to a benzene ring, which gives it a distinct chemical structure [2]. Its chemical formula is C_20_H_12_, and it has a molecular weight of 282.33 g/mol. 

Benzo[a]pyrene can be found in soil, water, and air, and it has the potential to accumulate in the food chain, especially in the fatty tissues of animals. Exposure to benzo[a]pyrene can happen through ingestion, inhalation, and skin contact [3], posing a potential threat to human health through food chain contamination and other environmental pathways [4]. Although B[a]P is strongly bound to the organic matter in soil and accumulates in the adipose tissue of plants and animals, it does not penetrate deeper soil layers. This is due to its low absorption by the plant’s root system [5].

GC-MS, LC-MS, and HPLC are among the chromatographic techniques used to monitor B[a]P in different matrices [6,7,8]. Chromatographic techniques offer several benefits. However, their complex sample preparation and need for skilled operators make them less suitable for on-site testing of numerous samples. In contrast, immunoassays are more attractive because of their high sensitivity, efficiency, and minimal operator expertise required [9]. Alternative approaches for benzo[a]pyrene detection are gaining acceptance among researchers. These approaches have several advantages, including speed, sensitivity, and cost-effectiveness. Immunoassays use antibodies to identify specific analytes in samples, including PAHs such as benzo[a]pyrene. The enzyme-linked immunosorbent tests (ELISA) are very specific, allowing for both qualitative and semi-quantitative analysis [9].

IgY is an antibody found in the blood of birds, reptiles, and some amphibians. It is the avian equivalent to mammalian IgG antibodies. The most prevalent antibody class in these species is IgY, which plays an essential role in their immunological responses. IgY antibodies are structurally similar to mammalian IgG antibodies, with two heavy chains and two light chains connected by disulfide links. They are members of the IgY subclass of immunoglobulins, which is further classified into subclasses (e.g., IgY1, IgY2) according to species. Chicken immunoglobulin Y (IgY) is a highly conserved equivalent to human immunoglobulin G (IgG) that has demonstrated efficacy and safety, particularly in animal models of human infectious illnesses. IgY is a low-cost, fast-acting antibody that can be generated in large quantities using egg-laying chickens with no adverse environmental effects and minimum infrastructure requirements [10]. IgY is an appropriate antibody for immunological studies involving mammalian serum because it does not interact with human anti-mouse IgG antibodies, activate the complement system, or bind to Fc receptors [11]. Because polyclonal antibodies can bind to many epitopes, they have a considerable advantage in identifying tiny compounds, according to research findings reported [12]. This feature makes them more applicable in small-molecule ELISAs, particularly where cross-reactivity with structurally related drugs is advantageous. PAbs are a popular option for a range of analytical applications since their fabrication method is also easier and less expensive. Due to immunological differences, IgY shows minimal cross-reactivity with mammalian IgG. As a low-cost antibody that can be produced through various methods, IgY is an appealing option for research and diagnostic purposes [13].

## 2. Materials and Methods

### 2.1. Material and Chemicals

NHS, N-hydroxy succinimide (Sigma-Aldrich, Darmstadt, Germany). EDC, 1-Ethyl-3-(3-dimethylaminopropyl) carbodiimide (Sigma-Aldrich, St. Louis, MO, USA). DMF, Dimethylformamide (Merck, Darmstadt, Germany). BSA, Bovine serum albumin (Sigma-Aldrich, Germany). OVA, Ovalbumin (Sigma-Aldrich, Germany). DMSO, Dimethyl sulfoxide (ACI Labscan, Bangkok, Thailand). Freund’s complete adjuvant (Sigma-Aldrich, Germany). Freund’s incomplete adjuvant (Sigma-Aldrich, Germany). HRP-IgY, Enzyme immunoassay-grade horseradish peroxidase-labelled goat anti-chicken immunoglobulin (Invitrogen, Rockford, IL, USA). Skim milk, O-phenylenediamine dihydrochloride, and OPD (Sigma-Aldrich, USA). Sulfuric acid, H_2_S0_4_ (J.T. Baker, Philadelphia, PA, USA). Sodium carbonate, Na_2_CO_3_ (Merck, Germany). Sodium Bicarbonate, NaHCO_3_ (Merck, Germany). Polysorbate 20, Tween-20 (Loba Chemie, Mumbai, India). Sodium chloride, NaCl (ACI Labscan, Thailand). Potassium chloride, KCl (Sigma-Aldrich, Germany). Disodium hydrogen phosphate, Na_2_HPO_4_ (ACI Labscan, Thailand). Potassium dihydrogen phosphate, KH_2_PO_4_ (Merck, Germany). Citric acid, C_6_H_8_O_7_ (Merck, Germany). TNBS, 2,4,6-Trinitrobenzenesulfonic acid (Sigma-Aldrich, USA). L-glutamic acid C_5_H_9_NO_4_. Methanol (J.T. Baker, USA). PEG 8000, Polyethylene Glycol 8000 (Bio Basic, Toronto, ON, Canada). Chrysene, Benzo[b]fluoranthene, Benzo[a]anthracene, and Benzo[k]fluoranthene were purchased from LGC Labor GmbH, Augsburg, Germany. Benzo[a]pyrene was purchased from Sigma-Aldrich, Missouri, USA. Pyrenebuteric acid was purchased from Sigma-Aldrich, Darmstadt, Germany. Benzo[a]pyrene diol epoxide was purchased from Toronto Research Chemicals, Toronto, ON, Canada. Acetonitrile (Avantor Inc., Radnor Township, PA, USA). Bio-Rad Protein Assay Dye Reagent Concentrate (Bio-Rad Laboratories, LifeScienceGroup, Hercules, CA, USA).

### 2.2. Methodology

#### 2.2.1. Immunogen Preparation for Immunization and Antigen Coating for Immunoassay Development

**Figure 1:**
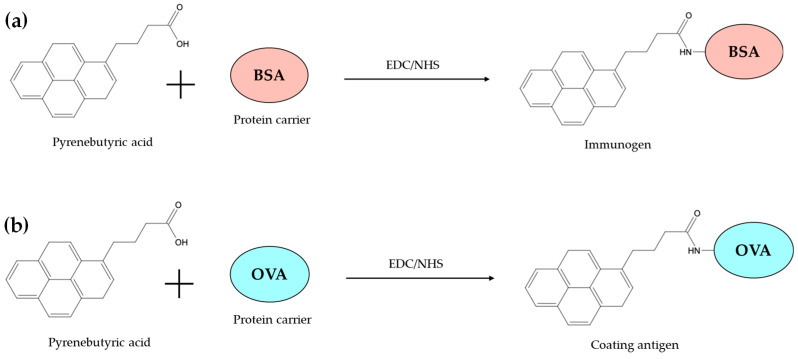
The synthesis technique of the immunogen (**a**) and coating antigen (**b**).

The synthesis technique of the coated antigen and immunogen was adjusted by Wu et al., 2022, and Meng et al., 2015 [7,14] as shown in Figure 1. The chemical pyrenebutyric acid (110 mg) is first reacted with 130 mg of 1-Ethyl-3-(3-dimethylaminopropyl) carbodiimide and 210 mg of N-hydroxysuccinimide, subsequently dissolved in 3 mL of Dimethylformamide. The solution will be gently stirred overnight at 4 °C, resulting in an activated solution. BSA (70 mg) was dissolved in PBS at pH 7.4 (9 mL) and then integrated into the activated solution, which was carefully transferred into a container and dialyzed with PBS at 4 °C for 5 days, with the dialysate changing every 12 h. The utilized immunogens were PyBA-BSA.

The coated antigens PyBA-OVA are synthesized in the same manner as the immunogen. In summary, 90 mg of OVA is dissolved in 12 mL PBS, and the resultant activated solution is introduced into three glass bottles holding OVA protein solution. The solutions are delicately agitated. The coating antigens are denoted as PyBA-OVA. The samples undergo dialysis with PBS at 4 °C for a duration of 5 days, with the dialysate being replaced every 12 h. The antigen is collected and stored in a refrigerator at 4 °C for future use. A UV-visible spectrophotometer will be employed to confirm antigen synthesis and assess hapten and protein ratios.

#### 2.2.2. Determination of Hapten Density in Immunogens and Coating Antigens

The 2,4,6-trinitrobenzenesulfonic acid (TNBS) assay is an indirect method for assessing hapten density [15]. TNBS accounts for surface amines before and after conjugation, with a decrease in surface lysines. The method finds the free amino groups of amino acids and peptides in column eluates. These groups react with each other to form trinitrophenyl (TNP) derivatives. 

TNBS reacts explicitly with primary amines to produce a yellow-colored product, visible at 420 nm. The hapten conjugated were suspended in 0.1 M NaHCO_3_, pH 8.0, and treated with TNBS (250 µL, 0.01% in 0.1 M NaHCO_3_). The reaction mixture’s final volume and final protein concentration, calculated as [Hapten conjugated], were 500 μL and 1 μM, respectively. The reaction was incubated at 37 °C for 2 h, and 100 µL of the solution was transferred to the UV 96-well plate. We conducted measurements of absorbance at 420 nm. We plotted the corresponding calibration curve with L-glutamic acid as the standard to determine the free amine concentration, [Amine]. The number of amines per protein was calculated using the expression: 


$$\mathrm{Number}\;\mathrm{of}\;\mathrm{amines}=\frac{\left[\mathrm{Amine}\right]}{\left[\text{Hapten-conjugated}\right]}$$

#### 2.2.3. Antibody Production and Characterization

Animal studies are carried out in compliance with the recommendations of the animal ethics committee. The production method of the coating antigen and immunogen was slightly modified [16]. Briefly, immunogens (PyBA-BSA) will be administered to 6-week-old hens. Immunogens are mixed with complete Freund’s adjuvant (CFA) and delivered subcutaneously at several sites in the hen’s breast for the initial immunization. Following that, immunogens emulsified with incomplete Freund’s adjuvant (FIA) were used to increase immunization every 14 days. To determine serum titer and sensitivity, an indirect competitive ELISA (ic-ELISA) is performed. For booster immunization, a hen with a high serum titer and sensitivity is chosen and inoculated with immunogens free of Freund’s adjuvant. Before collecting antibodies from egg yolks, the level of IgG antibody in serum will be measured 14 days, 28 days (second dosage), and 42 days (third dose) following the initial immunization. 

Chicken egg yolks are used to isolate IgY antibodies [17]. Briefly, after combining three dosages of egg yolks in PBS (pH 7.4), PEG 8000 is added to reach the necessary final concentration and vortexed until dissolved. The slurry is then rolled using a rolling mixer. The precipitate is removed by centrifuging the mixtures at 13,000× *g* for 20 min at 4 °C. PEG 8000 is added to the supernatant. The samples are blended using a rolling mixer. The mixtures are centrifuged again. The IgY-containing precipitate pellets are diluted in PBS using the same procedure as described previously and then precipitated again with PEG 8000. The pellets are filtered through a 0.45 um filter, dissolved in PBS, and stored at −20 °C. 

The concentration was measured using the Bradford protein assay, which estimates protein concentration based on absorbance. Briefly, prepare the dye reagent by diluting one part of the dye reagent concentrate with four parts of distilled water, resulting in a 1:5 dilution. Utilize the Whatman #1 filter to eliminate particles. The bovine serum albumin (BSA) at known concentrations (0, 0.1, 0.2, 0.3, 0.4, and 0.5 mg/mL), achieved by diluting a stock solution of BSA in distilled water, was used for the standard solution. Dilute samples at ratios of 1:10, 1:50, and 1:100 with water, and subsequently Dispense 10 µL of each standard into triplicate wells of the microtiter plate, then dispense 10 µL of each sample dilution into triplicate wells of the microtiter plate. Introduce 200 µL of the diluted dye reagent into each well. Incubate at ambient temperature for a minimum of 5 min. Assess absorbance at 595 nm.

The purity of the lgY antibody will be evaluated using SDS-PAGE and Coomassie blue staining. The protein profile of sodium dodecyl sulfate-polyacrylamide gel electrophoresis (SDS-PAGE) will be utilized to investigate IgY in egg yolk samples. SDS-PAGEs are carried out under both reducing and non-reducing conditions. SDS-PAGE will be performed using polyacrylamide gels. To determine total protein content, the protein concentration is first diluted. The substance is then mixed with the Laemmli loading buffer (with in/out 2-mercaptoethanol). The sample volume is loaded, and an IRIS11-stained protein ladder is utilized to compare molecular weights. SDS-PAGEs are performed in a tris/glycine/SDS running buffer for 60 min at 120 V using a Mini-PROTEAN^®^ Tetra System (Bio-Rad). The proteins were stained with Coomassie Brilliant Blue G-250 overnight, then discolored at room temperature. All gel pictures were inspected using a Bio-Rad ChemiDoc MP.

#### 2.2.4. Indirect Competitive ELISA Development

The Indirect Competitive ELISA method (16). The 96-well immunoassay plate will be coated with a coating antigen (PyBA-OVA) overnight at 4 °C. The plates are washed four times with PBST before being blocked for one hour with gelatin. In a distinct well (triplicate), the standards (Pyrenebutyric acid, Benzo[a]pyrene, Chrysene, Benzo[b]fluoranthene, Benzo[a]anthracene, Benzo[k]fluoranthene, and Benzo[a]pyrene diol epoxide) were introduced at varying concentrations (0–20 µg/mL) in skim milk PBST, followed by the antibody dilution and a 1 h incubation period. After blocking, the solution will be removed, and the mixture (antibody and standards) will be added to the plate and incubated for 1 h again. Plates will be washed four times before incubating for one hour with goat anti-chicken IgY-HRP diluted in PBST. It washes out four times more before adding the freshly produced OPD solution and incubating for a final 30 min before stopping the reaction with sulfuric acid. All incubations will take place in the dark. Finally, absorbance will be measured at 492 nm using a microplate reader.

#### 2.2.5. Assessment of Polyclonal Antibody

The IgY polyclonal antibody’s specificity, or capacity to identify other structurally related molecules, was assessed using IC_50_ values (µg/mL) to quantify the proportion of cross-reactivity. The IC_50_ values were used to determine the cross-reactivity percentage for each molecule, represented as a percentage, and determined the following way:

CR (%) = 100 × IC_50_ (value of hapten)/IC_50_ (value of competitor)

#### 2.2.6. Sample Preparation

The sample extraction was modified [7]. In short, three grams of homogenized material were deposited in a 15 mL conical tube, followed by 3 mL of PBS buffer, and agitated with a vortex mixer. Next, add 4 mL of ethyl acetate and 2 mL of acetonitrile, violently mix for 5 min using a vortexer at 2500 rpm at room temperature, and centrifuge at 5000× *g* for 5 min. Nitrogen was used to almost completely evaporate the supernatant. The residue was dissolved in 1 mL of methanol, agitated rapidly, and allowed to stand for 5 min before the supernatant was collected. We then completely evaporated the supernatant and dissolved it with 10% methanol in PBS pH 7.2 before testing it using ic-ELISA.

#### 2.2.7. Validation Test of ic-ELISA

The ELISA validation procedure was conducted as follows: grilled pork samples were analyzed using ic-ELISA. The limit of detection (LOD) and limit of quantification (LOQ) were determined based on the total number of blank samples. The recovery and intra-assay variability (CV) of spiked samples were analyzed repeatedly to assess precision and accuracy. The blank samples were spiked with three different amounts of B[a]P (0.1, 1, and 10 µg/mL), followed by three intra-batch tests for ELISA. The recovery and CV were computed using the following formulas: recovery (%) = (conc. measured/conc. spiked) × 100, and CV (%) = sample standard deviation/sample average × 100%.

## 3. Results and Discussion

### 3.1. Immunogen and Coating Antigen Characterization

#### Hapten Density of Immunogens and Coating Antigens

The quantity of haptens covalently bonded to the surface of a carrier molecule is essential for evaluating vaccine efficacy. Dialysis is utilized to purify the hapten-density materials. Table 1 shows the trinitrobenzene sulfonic acid (TNBS) technique revealed that the hapten density for BSA-PyBA and OVA-PyBA conjugates was 18.48 and 66.06, respectively.

### 3.2. Production of Antibody to Benzo[a]pyrene

#### 3.2.1. Preparation of Immunoglobulin Y from Egg Yolk and Assessment of Its Sensitivity and Specificity

**Figure 2:**
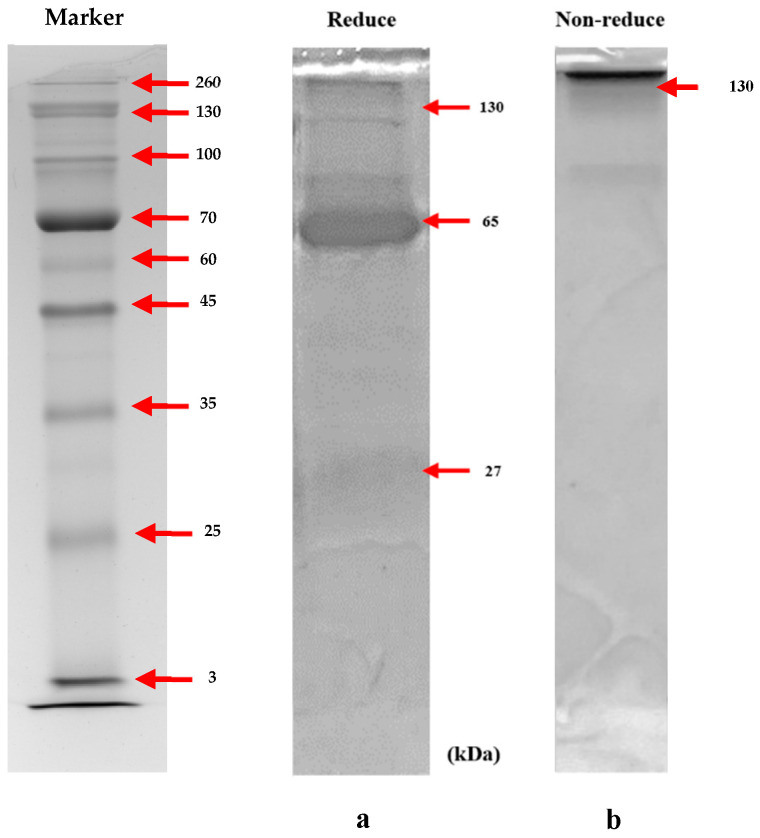
SDS-PAGE demonstrated the release of complete IgY from antibodies generated from egg yolk. (**a**) The substantial chains (27, 65, and 130 kDa under reducing circumstances, and (**b**) 130 kDa under non-reducing conditions) were identified utilizing a 10% resolving gel.

After the third immunization, eggs were collected, and the yolks were purified using PEG8000. The concentration, determined with the Bradford protein assay, is 12.57 mg/mL. The molecular weight and purity of the IgY antibody were then evaluated using sodium dodecyl sulfate–polyacrylamide gel electrophoresis (SDS–PAGE). The results from this analysis were crucial for assessing the water-soluble fractions after IgY extraction. According to the protein ladder, the heavy chains of the egg yolk antibody IgY displayed estimated molecular weights of 27, 65, and 130 kDa under reducing conditions and 130 kDa under non-reducing conditions. These observed molecular weight patterns matched the expected values (Figure 2). Through SDS–PAGE analysis, it was demonstrated that the purified fraction obtained from chromatography aligned with the anticipated sizes of IgY heavy chains. Thus, the presence of heavy chains at 27, 65, and 130 kDa was confirmed through validation via SDS-PAGE. In a recent study titled “Development of IgY-Based Indirect Competitive ELISA for the Detection of Fluoroquinolone,” the results were validated by SDS-PAGE, and the purified fraction matched the anticipated sizes of IgY heavy chains (27, 65, and 130 kDa) [18], and the water-soluble fractions obtained after IgY extraction were analyzed using SDS-PAGE (sodium dodecyl sulfate polyacrylamide gel electrophoresis). The molecular weight patterns corresponded to the expected molecular masses for the heavy and light chains, respectively. According to the protein ladder, the egg yolk antibody IgY has sizes of 65 and 27 kDa [10].

#### 3.2.2. Characterization of IgY Antibody Using Indirect Competitive ELISA

**Figure 3:**
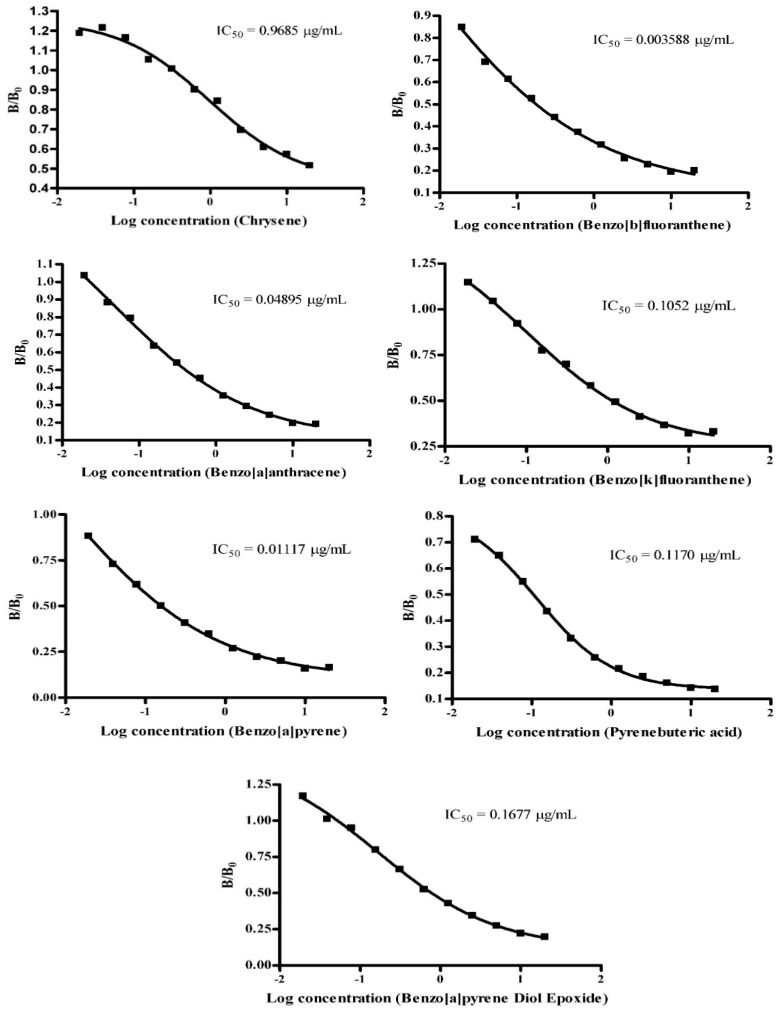
The inhibition curve of 6 PAHs and BPDE by using the PyBA–BSA activation of the antibody and PyBA–OVA as the coating antigen.

The ELISA protocol was established, and various parameters of the assay were fine-tuned using checkerboard analysis. Among these parameters, the concentration of the coating antigen was identified as having the most significant impact on assay sensitivity. Through optimization, it was determined that the optimal coating antigen concentration was 1 μg/mL. Similarly, the optimal concentration of the purified IgY antibody was determined to be 1/1000. The antibody’s specificity was tested against pyrene compounds using competitive assays, and the IC_50_ values (µg/mL) obtained were used to compute the proportion of cross-reactivity. Under optimal conditions, the ELISA method was utilized to generate a standard inhibitory curve for PyBA. By coating OVA-PyBA, eleven different concentrations of PAHs were introduced into the system. As a result, Figure 3. Shows the mean IC_50_ values for several PAHs, including Chrysene, Benzo[b]fluoranthene, Benzo[a]anthracene, Benzo[k]fluoranthene, B[a]P, PyBA, and BPDE, which were computed and found to be 0.97 μg/mL, 0.003 μg/mL, 0.05 μg/mL, 0.11 μg/mL, 0.01 μg/mL, 0.12 μg/mL, and 0.17 μg/mL, respectively.

### 3.3. Antibody Specificity

Competitive tests were employed to evaluate the antibody’s specificity towards six PAH groups and BPDE. The IC_50_ values (μg/mL) were utilized to determine the percentage of cross-reactivity. The results were utilized to calculate the cross-reactivity % of each chemical with B[a]P. Table 2 displays the mean data derived from two separate experimental trials, each consisting of duplicates. To determine antibody specificity, six PAH derivatives—PyBA, B[a]P, Chrysene, Benzo[b]fluoranthene, Benzo[a]anthracene, and Benzo[k]fluoranthene—were assessed in the experiment to compete for binding with PyBA. The results demonstrated that the antibody exhibited a notable affinity for Chrysene (1.15%), Benzo[b]fluoranthene (311.32%), Benzo[k]fluoranthene (10.62%), Benzo[a]anthracene (22.82%), and PyBA (9.55%). Nonetheless, its affinity for B[a]P persisted at 100%. The cross-reactivity percentage (%CR) for Benzo[a]pyrene diol epoxide was determined to be 6.66%. Prior research established that the specificity of the developed ic-ELISA for pyrenebutyric acid (PYR) monoclonal antibody (mAb) was evaluated by cross-reactivity. Standards containing 16 PAHs were tested for cross-reactivity with the 4D6 mAb. The monoclonal antibody exhibited a robust binding affinity for PYR and BaP at 100% and 38%, respectively, a modest affinity for fluoranthene (8%), and little cross-reactivity with other polycyclic aromatic hydrocarbons (<1%) [7].

### 3.4. Validation of ELISA for B[a]P Detection in Grilled Pork Samples

#### 3.4.1. Organic Solvents on Antibody

**Figure 4:**
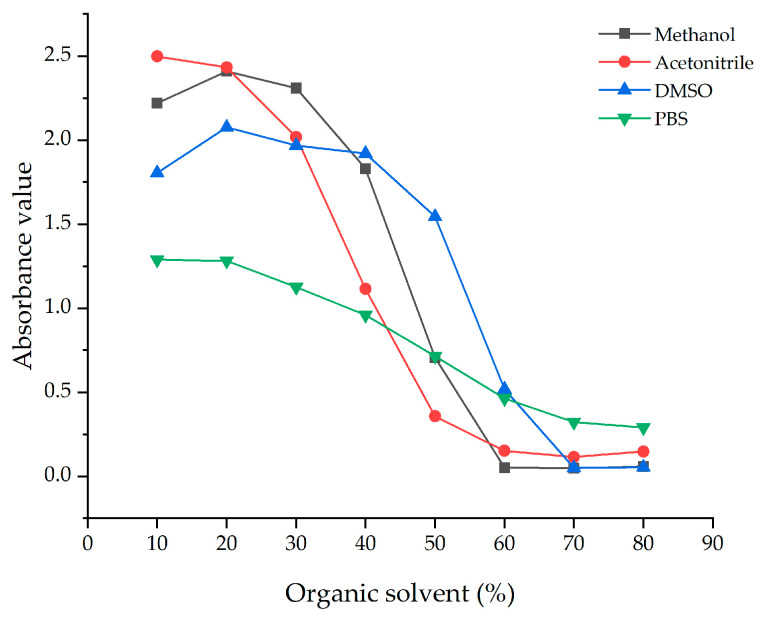
A comparison of organic solvents.

PAHs are hydrophobic, lipophilic compounds usually dissolved in organic solvents. It has been observed that organic solvents can affect the parameters of the ELISA [19]. Therefore, methanol, acetonitrile, and DMSO were chosen for examination in this experiment. Organic solvents introduced to the ELISA reaction system should have minimal effect on the antigen-antibody binding response. Therefore, it was crucial to examine the binding properties across different concentrations of organic solvents. Organic solvents with varying volume fractions (10%, 20%, 30%, 40%, 50%, 60%, 70%, and 80%) in PBS buffer were used as standard diluents. Figure 4. shows absorbance changes were minimal at methanol and DMSO concentrations of 10%, 20%, and 30%, respectively. While acetonitrile showed considerable absorbance changes. In the previous work, the effect of DMF and DMSO concentrations on ELISA sensitivity was studied. When the volume fractions of DMF and DMSO ranged from 10% to 40%, there was a notable variation in sensitivity. Conversely, a 20% DMF-PBS solution exhibited the lowest IC_50_ value, significantly enhancing the sensitivity of the ic-ELISA [7]. The use of organic cosolvents to enhance antibody affinity and specificity has been previously discussed [20]. As a result, 20% DMSO was chosen as the standard diluent.

#### 3.4.2. Recovery Study for ELISA in Matrix

Matrix interference is a common occurrence in ELISA recovery studies. This study demonstrates that simple dilution significantly mitigated matrix effects. Table 3. presents the recovery results for grilled pork samples spiked at concentrations of 1.00, 0.10, and 0.01 μg/mL. The intra-assay recoveries for grilled pork at spiked doses of B[a]P were 128.75–143.11%, 95.60–96.15%, and 74.99–88.89%, respectively. Consequently, the B[a]P was recovered effectively and consistently using this extraction method. A prior study developed a monoclonal antibody for the detection of pyrenebutyric acid (PYR) and benzo[a]pyrene (BaP) in aquatic samples. A reliable ic-ELISA method was established and employed to quantify PYR and BaP residues in fish and shrimp. The recovery rates of PYR and BaP in aquatic samples ranged from 81.5% to 101.9% and from 84.9% to 94.0%, respectively [7]. This study demonstrated that the heterologous ELISA for detecting B[a]P in grilled pork samples exhibited high sensitivity but showed significant cross-reactivity with benzo[b]fluoranthene, a structurally similar PAH. This highlights a limitation in distinguishing B[a]P from other PAHs in complex mixtures. The term “heterologous ELISA” denotes an indirect competitive ELISA system that considers variations in hapten structure, linker attachment site, or bridge characteristics. The differences frequently lead to diminished efficacy of antibody detection for the coating antigen relative to the target compound, thereby rendering it an appropriate method for identifying small molecules in immunoassays [20,21].

In comparison to other studies on the immunoassay of B[a]P or other PAHs, nearly all investigations employed monoclonal antibodies to create immunoassays for the identification of compounds in food [7] and environmental samples [14,19,22]. In this study, the antibody was polyclonal, and the IgY antibody was used for the first time to create an immunoassay for detecting benzo[a]pyrene. The ELISA had a higher IC_50_ value than the other immunoassays, but it still provided acceptable results. The IC_10_ and IC_90_ values were 0.00124 μg/mL and 0.1 μg/mL, respectively. The limit of detection was established at IC_10_. The European Union has reduced the maximum residue limits (MRL) for smoked foods to 2 μg/kg. The permissible total concentration of PAH4 (benzo[a]pyrene, benzo[b]fluoranthene, benzo[a]anthracene, and chrysene) remains at 12 μg/kg. The immunoassays demonstrated overall effectiveness in detecting quinolone antibiotic residues; however, certain assays exhibited reduced efficacy relative to conventional chromatography techniques. Additionally, several immunoassay techniques showed high sensitivity, indicating that the immunoassay approach can be comparable to conventional methods.

## 4. Conclusions

This study developed the antigen PyBA-BSA and utilized it to prepare a sensitive polyclonal immunoglobulin Y antibody, which was deployed for the first time in an immunoassay for detecting benzo[a]pyrene. The IgY had a strong affinity for B[a]P (100%). The DMSO concentration for extraction may be utilized to assess B[a]P residue at a concentration of 20% in the extract solution. The IC_10_ of B[a]P in the grilled pork sample was 1.24 µg/kg. The ic-ELISA approach developed in this work has shown efficacy in detecting B[a]P in grilled pork samples, filling the gap of an existing ELISA technique. This immunoassay represents a significant advancement in environmental analytical chemistry, providing a cost-effective and accessible method for detecting B[a]P, therefore aiding in the safeguarding of human health and the environment.

## Figures and Tables

**Table 1:** Hapten density of immunogens and coating antigen.

	Haptens Conjugation ^1^	Haptens Density (Number of Amines/Protein)
Immunogens	BSA-PyBA	18.48
Coating antigen	OVA-PyBA	66.08

^1^ A 0.1 M sodium bicarbonate solution with a pH of 8.0 and a hapten concentration of 890 µg/mL was prepared. This solution was then reacted with 0.01% TNBS, incubated, and the absorbance was measured at 420 nm using a spectrophotometer.

**Table 2:** In this ic-ELISA analysis, the IC_50_ and percentage of cross-reactivities (%CR) of PyBA to different pyrenes were determined.

Compounds	Structure	Coating Antigen	IC_50_ (µg/mL)	Cross-Reactivity (%)
Chrysene	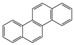	OVA-PyBA	0.97	1.15
Benzo[b]fluoranthene	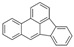	OVA-PyBA	0.003	311.32
Benzo[a]anthracene	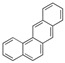	OVA-PyBA	0.05	22.82
Benzo[k]fluoranthene	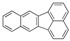	OVA-PyBA	0.11	10.62
Benzo[a]pyrene	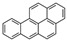	OVA-PyBA	0.01	100.00
Pyrenebuteric acid	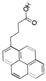	OVA-PyBA	0.12	9.55
Benzo[a]pyrene Diol Epoxide	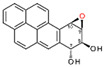	OVA-Prybar	0.17	6.66

**Table 3:** The percentage of recovery (%R) of different concentrations of Benzo[a]pyrene in various matrices, including the mean recoveries and coefficient of variation (CV) in matrices extracted from grilled pork.

Analyte	Spike Conc. (μg/mL)	Matrix	
		Pork	
		R (%)	CV (%)
Benzo[a]pyrene	10.00	128.75–143.11	7.47
	1.00	95.60–96.15	0.39
	0.10	74.99–88.89	3.17

## Data Availability

Data are contained within the article.
